# A First-Stage Approximation to Identify New Imprinted Genes through Sequence Analysis of Its Coding Regions

**DOI:** 10.1155/2009/549387

**Published:** 2009-04-08

**Authors:** Elias Daura-Oller, Maria Cabré, Miguel A. Montero, José L. Paternáin, Antoni Romeu

**Affiliations:** Biochemistry and Biotechnology Department, Faculty of Chemistry, Rovira i Virgili University (URV), c/Marcel-li Domingo, s/n. Campus Sescelades, 43007 Tarragona, Spain

## Abstract

In the present study, a positive training set of 30 known human imprinted gene coding regions are compared with a set of 72 randomly sampled human nonimprinted gene coding regions (negative training set) to identify genomic features common to human imprinted genes. The most important feature of the present work is its ability to use multivariate analysis to look at variation, at coding region DNA level, among imprinted and non-imprinted genes. There is a force affecting genomic parameters that appears through the use of the appropriate multivariate methods (principle components analysis (PCA) and quadratic discriminant analysis (QDA)) to analyse quantitative genomic data. We show that variables, such as CG content, [bp]% CpG islands, [bp]% Large Tandem Repeats, and [bp]% Simple Repeats, are able to distinguish coding regions of human imprinted genes.

## 1. Introduction

Genomic imprinting is an epigenetic modification of dispersed regions of the genome depending on their exposure to the maternal or paternal germline. This results in differential expression of only one of the two alleles depending on the parent of origin. Allele-specific CpG methylation, histone acetylation, asynchronous DNA replication, and chromatin condensation are all associated with imprinted loci [1].

Recently, the question of whether imprinted genes have sequence characteristics that distinguish them from non-imprinted genes is drawing the attention of several research groups. Such structural differences may elucidate the mechanisms leading to allele-specific expression of imprinted genes [2]. Greally [3] found that the main sequence characteristic of human imprinted genes is a lower incidence of short interspersed nuclear elements. For tandem repeats and CpG islands, there is accumulating evidence correlating these elements and genomic imprinting. Accordingly, some authors [4–7] suggested using these sequence features as a search tool for imprinted genes.

Identifying imprinted genes experimentally is challenging because the monoallelic expression of an imprinted gene may occur only in one of possibly several isoforms, only in particular tissues, or only at particular stages of development. Many autosomal genes are imprinted only in specific tissues or cell types, including GRB10 [8], Igf2/H19 [9], UBE3A [10], ATP10A (formerly ATP10C) [11], and KCNQ1 [12].

Consequently, in the absence of any method for prioritising genes, an average of 100 genes must be examined before a new imprinted gene can be identified. Indeed, experimental identification of human imprinted genes to date has been slow. To date, only ~60 human imprinted genes have been identified.

For this reason, the application of sequence analysis approaches to genome-wide screening of human genes, which can be ranked to identify those with a sequence composition suggestive of imprinting, is very useful.

To date, imprinted genes are predicted using a wide range of genomic features and sophisticated strategies and methodologies [13–16], but no simple sequence patterns and models are known to accurately distinguish imprinted genes from non-imprinted ones. But even so, a simple approach would be potentially valuable for directing laboratory work in a first stage.

We are concerned with identifying possible candidate imprinted genes to allow their imprinting status to be determined experimentally. For this reason, human gene coding region features are considered further with a view to developing an approximation to a first-stage screening and classifying genes into imprinted and non-imprinted candidate groups. This study uses statistical approaches for a first discrimination between imprinted and non-imprinted genes based on the currently available coding region sequences.

## 2. Materials and Methods

A positive training set of 30 human genes (Table 1) that showed imprinting effects were selected for analysis from the Catalogue of Imprinted Genes (http://igc.otago.ac.nz/home.html). A negative training set of 72 randomly selected control genes and a test set of 31 predicted imprinted genes were compiled from the recent literature [16] and were collected from the NCBI nucleotide database (http://www.ncbi.nlm.nih.gov/). See supplementary data for more details about these genes used in this study.

The sequence characteristics of the coding regions of each gene were examined in the analysis. These regions are the portions of a gene or an mRNA which actually code for a protein.

For CpG dinucleotide analysis, we used the NEWCPGREPORT program (http://mobyle.pasteur.fr/cgibin/portal.py?form=newcpgreport), and the total number of CpG islands was counted. For the repeat element analysis, the Repeat Masker program (http://www.repeatmasker.org/cgi-bin/WEBRepeatMasker) was used, and for tandem repeat analysis, the ETANDEM program (http://mobyle.pasteur.fr/cgi-bin/ MobylePortal/portal.py?form=etandem) was used. All classes of repeat elements output from Repeat Masker were collected. We used ETANDEM to obtain numbers of tandem repeat elements ranging from 5 bp to 100 bp. The Wilbur and Lipman pairwise sequence alignment method, implemented in the MegaAlign program of the DNAstar Sequence Analysis software (Lasergene v8.0; http://www.dnastar.com) used to align sequences of Large Tandem Repeats identified in imprinted genes.

Principal component analysis (PCA) and quadratic discriminant analysis (QDA) models of the [bp]% sequence characteristics data were performed using the Minitab software [17].

PCA analysis is a multivariate statistical technique. The central idea of PCA is to reduce the dimensionality of a data set that presents a large number of interrelated variables, while retaining as much as possible the variation present in the data set. PCA can search the data for qualitative and quantitative distinctions in situations where the number of data available is too large.

The purpose of the Quadratic Discriminant Analysis is to predict membership of a group from a set of predictor variables (the sequence characteristics). The discriminant is the quadratic combination of the predictor variables that best predicts group membership, allowing each gene to be classified into either imprinted or control groups on the basis of its sequence characteristics.

The performance of the classification was assessed using internal and external validation methods according to our software capabilities.

With the QDA model, we used an internal validation method called *cross-validation* [18]. This method uses the training set to check the model. Here, the training set is divided in several segments. One segment is reserved to corroborate the results, and the rest of them are used to build the model.

This process is repeated as many times as segments you have, and every time one of these segments is out of the calibration, and the other ones are used to build the model. Finally, all the segments are used to both build and validate the model.

With the PCA model, we used the external validation *test set* method. The number of elements of this set must be large (at least 25% of the training set size), and it must be independent of the training set, but also this test set must represent the training set. The imprinted status of the test set is known, so it is possible to assess the PCA model using different elements that the ones used to build the model.

## 3. Results and Discussion

Recently, Ke et al. [14] found significant statistical differences between some sequence descriptors of human imprinted and control gene coding regions. These significant variables in their regression model were the Simple and Large Tandem Repeats, GC content, CpG islands, and short interspersed nuclear elements.

Taking into account this fact, we considered these descriptors (variables) as the most relevant ones for our study. So, the [bp]% genomic sequence characteristics of GC content, CpG islands, simple repeats (SR), large tandem repeats (LTR) and SINEs of all imprinted and non-imprinted coding region sequences were calculated.

Before applying the pattern recognition methods, each calculated descriptor was autoscaled. In the autoscaling method, each variable is scaled to a mean of zero and a standard deviation of unity. This method is very important because each variable is weighted equally, and this provides a measure of the ability of a descriptor to discriminate classes of compounds [19]. With this method, we can compare all descriptors at the same level.

Firstly, we started applying the PCA technique. After several PCA analyses, the best separation was obtained by using the following descriptors: GC content, [bp]% CpG islands, [bp]% Simple Repeats and [bp]% Large Tandem Repeats. This suggests that in this case, the other variables are not significant for the classification of the coding regions studied.

**Figure 1:**
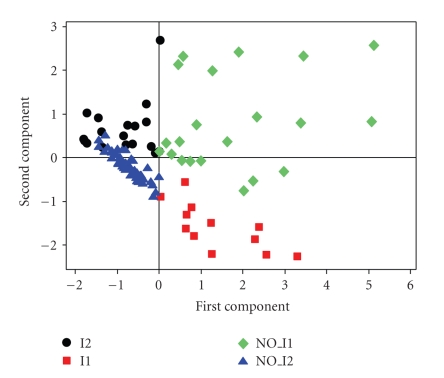
The separation of the training set into four groups: I1, I2, NO_I1 and NO_I2. Notice that both PCs are responsible for the separation.

The PCA results show that the first component (PC1) is responsible for 49.6% of the variance of the data. Considering the first (PC1) and second (PC2) components, the accumulated variance increases to 72%. Figure 1 shows that both PC1 and PC2 are in fact responsible for the discrimination between imprinted (two groups: I1 and I2) and non-imprinted (two groups: NO_I1 and NO_I2) genes. PC1 and PC2 can be represented by the following equations, that in fact form the PCA pattern recognition model:

(1) $$\begin{array}{ll} \text{PC}1 & =\;0.535\;[\text{GC}\;\;\text{content}]+0.511\;[[\text{bp}]\%\;\text{CpG}\;\;\text{islands}]\\ & \;+0.521\;[[\text{bp}]\%\;\text{LTR}]+0.426\;[[\text{bp}]\%\;\text{SR}],\\ \text{PC}2 & =-0.425\;[\text{GC}\;\;\text{content}]-0.467\;[[\text{bp}]\%\;\text{CpG}\;\;\text{islands}]\\ & \;+0.313\;[[\text{bp}]\%\;\text{LTR}]+0.71\;[[\text{bp}]\%\;\text{SR}]. \end{array}$$

**Figure 2:**
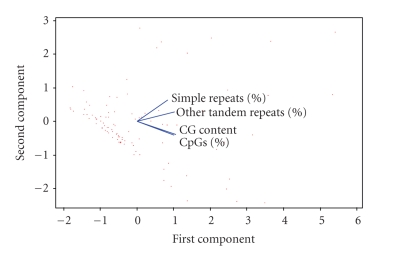
Plot of the loading values of the selected variables used in the training set.

From Figure 2 and (1), we can see that the imprinted group I1 has large values for GC content and [bp]% CpG islands and a major content of [bp]% LTR compared with the I2 group. The imprinted group I2 has small values for GC content and [bp]% CpG islands and a major content of [bp]% SR.

On the other hand, we can see that the major part of non-imprinted genes, the NO_I2 group, has small values for [bp]% SR and [bp]% LTR, and the NO_I1 group has large values for the same both descriptors. It is clear that there are four coding region groups, and each one is located in practically one specific quadrant of the *XY* axes.

Genomic sequence characteristics of a total of 22544 bp from the coding sequences of 12 (I1 group) imprinted genes were compared to those of 66959 bp of coding sequences of 18 (I2 group) imprinted genes (Table 2) in order to carry out a deep study of the most relevant imprinted descriptors. The average number of CpG islands was higher in I1 group (1.8) than in I2 group (0.4). The frequency of G + C was also higher in I1 genes (62%) than in I2 ones (45%). Moreover, the average number of the ratio [bp]% LTR/[bp]% *coding sequence* coefficient is higher in the I1 group (I1) than in I2 (0.03). Note that these results are in good agreement with the loadings of the PCA model.

We found an obvious functional difference between I1 and I2 groups in terms of expression pattern. We observed maternal expression for 67% of the I1 imprinted genes and paternal expression for 61% of the I2 imprinted genes.

Moreover, other important observation is that all the Large Tandem Repeats of the I1 group genes are inside CpG islands while this fact is not observed in the I2 group. These results agree with those of Meguro et al. [11]: the CpG islands of imprinted genes contain some special DNA elements that distinguish them from CpG islands of biallelically expressed genes.

To identify sequence fingerprints and similarities among Large Tandem Repeats in the two imprinted groups, we used the Wilbur and Lipman pairwise sequence alignment method (see supplementary data for details). The I1 sequences group is quite consistent; all sequences are rich in GC content, and the similarity index of the aligned fragments ranges from 60 to 100%. In contrast, the sequences of the I2 group are longer, more heterogenous in terms of nucleotide composition; in some of them, the presence of a polyA motif could be empathised. The I2 sequence repeats show a much more wide range of similarity index. In addition, because of some significant differences in nucleotide composition between members of I2 sequences, some I2 sequence pairs could not to be aligned. From this analysis, we can conclude that these two Large Tandem Repeats: GC-motifs (in I1 group) and AT-motifs (in I2 group) are highly conserved sequence patterns across their respective coding regions.

Then, we built a new model using another statistical technique: the quadratic discriminant analysis (QDA). QDA is also closely related to principal component analysis (PCA) in that both look for combinations of variables which best explain the data. QDA explicitly attempts to model the difference between the classes of data (supervised pattern recognition). PCA, on the other hand, does not take into account any difference in class (nonsupervised pattern recognition).

Table 3 shows the results of the QDA classification model. The total percentage of correct classification was 93%, and the proportions for each group are 100% (I2), 92% (I1), 90% (NO_I1) and 92% (NO_I2).

After the employing of QDA and PCA methods, we proceeded to the validation of their respective classification models.

The *cross-validation* approach was used to validate the QDA model. The total percentage of correct classification was 83% (Table 3). Therefore, this result confirms the existence of four groups between the coding regions characteristics.

On the other hand, the *test set* approach was used to validate the PCA model. We decided to apply the PCA model to a series of new predicted imprinted genes whose imprinting status was predicted by other methodologies [16] but it is still not experimentally proved. In this way, apart from the construction of a representative test set, we could compare our PCA results with the ones of Luedi et al. [16].

To form a randomly test set, we did a full-text mining search with all Luedi's predicted gene names across the publication data of the *Nutrigenomics Database* (http://133.11.220.243/nutdb.html). After that, we formed a test group of 31 supposed imprinted genes related to nutrigenomics in humans (Table 4). It is important to emphasise that these possible imprinted genes are related with dietary factors known to influence DNA methylation as alcohol, folate, zinc, and cadmium. We thought that this fact may be interesting for future nutrigenomic work.

We calculated the genomic sequence characteristics of the 31 coding regions, and then we checked if our PCA pattern recognition model could classify them as imprinted genes, too.

**Figure 3:**
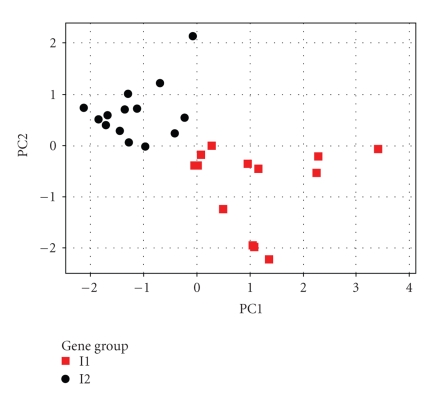
Scores for the predicted imprinted genes.

Figure 3 shows the results of the PCA calculations for the first (PC1) and second (PC2) principal components. Before carrying out the prediction calculations, the descriptors were also autoscaled as previously. We found that 27 of the 31 genes were classified in the two correct imprinted quadrants (84%) by the PCA model. The GFI1, HSPA6, HOXD9, PITX2, PTPRN2, GADD45G, GATA3, NRGN, F10, JAG2, GATA6, ELA2, and ZNF42 genes are classified in the I1 imprinted group. The I2 imprinted groups are formed by EFNA4, BCL2L11, PER2, PPARG, POLR2H, TLL1, NDUFSA4, ITGB8, CDK6, AKR1C2, KLRF1, KLRC3, POU4F1, and SFRS2 genes.

Therefore, taking together these results and the ones of Luedi et al., we can suggest these 27 genes as good candidates for an experimental imprinting determination.

## 4. Conclusions

The most important feature of the present work is its ability to use multivariate analysis to look at variation, at coding region DNA level, among imprinted and non-imprinted genes. There is a force affecting genomic parameters that appears through the use of the appropriate multivariate methods (principle components analysis (PCA) and quadratic discriminant analysis (QDA) to analyse quantitative genomic data. We show that variables, such as, CG content, [bp]% CpG islands, [bp]% Large Tandem Repeats, and [bp]% Simple Repeats are able to distinguish human coding region imprinted genes.

We know that a conclusive assessment of prediction methods for imprinted genes is problematic due to the small number of affected genes, their clustering in small genomic regions, and the difficulty of experimental validation.

However, we think that the application of this PCA sequence analysis approach to genome-wide screening of human genes, which can be ranked to identify those with a sequence composition suggestive of imprinting, is potentially valuable for a first-stage approximation directing follow-up laboratory work.

Clearly an approach like this can be further refined and the resolution improved as more imprinted genes are identified and confirmed and the genome sequencing is completed.

## Supplementary Material

Supplementary material containing lists of genes used in the study: a negative training set of 72 randomly selected control genes and a test set of 31 predicted imprinted genes; a table with the relevant calculated features; results of Wilbur and Lipman pairwise sequence alignment method.

## Figures and Tables

**Table 1:** List of imprinted genes classified by expression.

Name	Band	Expression
TP73	1p36	M
LRRTM1	2p12	P
NAP1L5	4q22	P
PRIM2	6p12	M
PLAGL1	6q24	P
HYMAI	6q24	P
PEG10	7q21	P
PON1	7q21	P
CALCR	7q21	M
PPP1R9A	7q21	M
MEST	7q32	P
COPG2	7q32	P
CPA4	7q32	M
KLF14	7q32	M
KCNK9	8q24	M
INPP5F_V2	10q26	P
KCNQ1	11p15	M
IGF2AS	11p15	P
SMPD1	11p15	M
IGF2	11p15	P
ZNF215	11p15	M
H19	11p15	M
SLC22A18	11p15	M
PHLDA2	11p15	M
NDN	15q11	P
MKRN3	15q11	P
MAGEL2	15q11	P
UBE3A	15q12	M
TCEB3C	18q21	M
NNAT	20q11	P

**Table 2:** The number of large tandem repeats (LTR), CpG islands, and GC content in coding sequences of imprinted genes.

I1 group	Lenght	CG content	Number CpG islands	Number LTR	Size count	Consensus
TP73	2234	64.6	3	0	—	—
LRRTM1	2217	58.4	2	1	24_7	ctgccgaaccacaccttccaggac
KLF14	1383	66.8	2	1	18_9	cggcgcgcccgccgcctc
KCNK9	1303	60.1	2	0	—	—
KCNQ1	3262	63.4	1	1	30_4	cgcggccgccgccccgggccccgcgccccc
IGF2AS	2056	64	1	0	—	—
SMPD1	2473	59.8	1	1	6_9	cgctgg
IGF2	1356	63.7	3	1	14_18	tccccccctctctc
SLC22A18	1549	65	1	0	—	—
PHLDA2	937	61.7	1	1	9_14	ccgcgccct
NDN	1897	52.3	2	1	57_4	cccaggcccacaacgccccgggcgccccgaaggcggttccgccggccgcggccccgg
TCEB3C	1877	64.7	2	0	—	—

I2 group	Lenght	CG content	Number CpG islands	Number LTR	Size count	Consensus

NAP1L5	1912	42.9	0	1	12_7	ggaggaggagga
PRIM2	2353	40.7	0	0	—	—
PLAGL1	4354	46.9	1	1	25_3	atcttacaaaaaaaaaaaaaaaaaa
HYMAI	5005	42.1	1	1	13_7	tatatatatataa
PEG10	6628	44.7	2	2	42_3 12_4	agaagctctcagaggagaacaacaaccttcgagagcaggtgg/ccgccgcctcca
PON1	2395	41.3	0	0	—	—
CALCR	3470	40.4	0	0	—	—
PPP1R9A	9705	39.9	0	1	5_8	ttttc
MEST	2507	45.1	1	2	42_4 23_3	ggcggctgcggctgccgcgcccggtgctgcccagcgctgcgg/caaaaaaaaaaaaaaaaaaaaaa
COPG2	3365	43.1	0	0	—	—
CPA4	2807	48.9	1	0	—	—
INPP5F_V2	4955	43.5	1	0	—	—
ZNF215	3658	40.4	1	2	84_3 84_3	tattcgacatcaaaaaattcatactgaagcgaaggcctataaatgcaataaatgtgggaaagccttcagccgaagtgcagacct/aaaactgcatactggagataagtcctgaaaatgtaaaaaatgtaggaaaaccttcaaccggagttcagaacttatttaacatca
H19	2615	55.9	0	2	8_10 20_4	ggggggga/ctttttcttcttcctccttt
MKRN3	3107	48	0	1	29_5	ttaaaaattatatatataagaatataaaa
MAGEL2	2294	53.7	0	2	36_7 21_3	cgggccctgagtgtctgggagggcccaagcacctcc/ggcctcctcaaaagagcgcag
UBE3A	4491	36.7	0	1	10_7	aaaacaaaaa
NNAT	1338	56.5	0	0	—	—

**Table 3:** Classification obtained with the QDA analysis.

Group	I2	I1	NO_I1	NO_I2
count	18	12	21	51

Summary of classification				

True group				

Put into group	I2	I1	NO_I1	NO_I2
I2	18	0	2	3
I1	0	11	0	1
NO_I1	0	0	19	0
NO_I2	0	1	0	47
Total *N*	18	12	21	51
*N* correct	18	11	19	47
Proportion	1,00	0,92	0,90	0,92

*N* = 102; *N* correct = 95; proportion correct = 0,93; proportion correct with cross-validation = 0.833.

**Table 4:** List of 31 genes from the test group.

Gene	Expression	Lenght	Chromosome
GFI1	P	2784	1
EFNA4	M	1276	1
HSPA6	M	2664	1
SHC1	M	1752	1
CYP1B1	P	5128	2
SIX3	P	1926	2
OTX1	M	2176	2

BCL2L11	P	3422	2
HOXD9	M	2089	2
PER2	M	6219	2
PPARG	P	1883	3

POLR2H	M	821	3
PITX2	P	2122	4
TLL1	P	6654	4

NDUFS4	P	668	5
ITGB8	M	8787	7
CDK6	M	11611	7
PTPRN2	M	4767	7

GADD45G	P	1078	9
AKR1C2	P	1663	10
GATA3	P	3070	10
NRGN	P	1295	11
KLRF1	P	1242	12
KLRC3	P	1042	12
POU4F1	M	5015	13
F10	M	1560	13
JAG2	M	5077	14
SFRS2	M	2923	17
GATA6	M	3494	18
ELA2	M	938	19
ZNF42	M	2620	19
