# Supplementary material for: A First-Stage Approximation to Identify New Imprinted Genes through Sequence Analysis of Its Coding Regions
Source: Comp Funct Genomics. 2009 Apr 8;2009:549387. doi: 10.1155/2009/549387 (PMC2666875; doi:10.1155/2009/549387)
Supplement: Supplementary file 1 — Supplementary material containing lists of genes used in the study: a negative training set of 72 randomly selected control genes and a test set of 31 predicted imprinted genes; a table with the relevant calculated features; results of Wilbur and Lipman pairwise sequence alignment method. [file 549387.f1.doc]

**Supplementary Data**

**A first stage approximation to identify new imprinted genes through sequence analysis of its coding regions**

*E. Daura-Oller,* *Maria Cabré, Miguel A. Montero, José L. Paternáin, A. Romeu*

*Biochemistry and Biotechnology Department, Faculty of Chemistry, Rovira i Virgili*

*University (URV), c/ Marcel·li Domingo, s/n. Campus Sescelades, 43007 Tarragona, Spain*

| **Imprinted Genes List** | | |  |  |
| --- | --- | --- | --- | --- |
|  | |  |  |  |
| Name | | Band | Expression | Reference |
|  | |  |  |  |
| TP73 | | 1p36 | M | Kaghad et al. (1997); Mai et al. (1998); Cai et al. (2000) |
| LRRTM1 | | 2p12 | P | Francks C et al, (2007) |
| NAP1L5 | | 4q22 | P | Wood AJ et al, (2007); Smith RJ et al, (2003) |
| PRIM2 | | 6p12 | M | Pant PV et al, (2006) |
| PLAGL1 | | 6q24 | P | Kamiya et al. (2000) |
| HYMAI | | 6q24 | P | Arima et al. (2000) |
| PEG10 | | 7q21 | P | Ono et al. (2001) |
| PON1 | | 7q21 | P | Okita C et al, (2003) |
| CALCR | | 7q21 | M | Okita et al. (2003) |
| PPP1R9A | | 7q21 | M | Nakabayashi et al. (2004) |
| MEST | | 7q32 | P | Kobayashi et al. (1997) |
| COPG2 | | 7q32 | P | Yamasaki K et al, (2000) |
| CPA4 | | 7q32 | M | Kayashima et al. (2003) |
| KLF14 | | 7q32 | M | Parker-Katiraee L et al, (2007) |
| KCNK9 | | 8q24 | M | Ruf D et al, (2007) |
| INPP5F_V2 | | 10q26 | P | Wood AJ et al, (2007); Smith RJ et al, (2003) |
| KCNQ1 | | 11p15 | M | Lee et al. (1997) |
| IGF2AS | | 11p15 | P | Okutsu et al. (2000) |
| SMPD1 | | 11p15 | M | Simonaro et al. (2006) |
| IGF2 | | 11p15 | P | Ogawa et al. (1993a); Ohlsson et al. (1993); Rainier et al. (1993) |
| ZNF215 | | 11p15 | M | Alders et al. (2000) |
| H19 | | 11p15 | M | Zhang and Tycko (1992) |
| SLC22A18 | | 11p15 | M | Cooper et al. (1998); Bajaj et al. (2004) |
| PHLDA2 | | 11p15 | M | Qian et al. (1997) |
| NDN | | 15q11 | P | MacDonald and Wevrick (1997); Jay et al. (1997) |
| MKRN3 | | 15q11 | P | Driscoll et al. (1992); Glenn et al. (1993, 1997); Jong et al. (1999) |
| MAGEL2 | | 15q11 | P | Boccaccio et al. (1999); Lee et al. (2000) |
| UBE3A | | 15q12 | M | Rougeulle et al. (1997); Vu and Hoffman (1997) |
| TCEB3C | | 18q21 | M | Strichman-Almashanu et al. (2001) |
| NNAT | | 20q11 | P | Evans et al. (2001) |
|  | |  |  |  |
|  | |  |  |  |
| **Non-Imprinted Genes List** | | |  |  |
|  |  | |  |  |
| Name | Band | | Expression | Reference |
|  |  | |  |  |
| NGFB | 1p13 | | X | P. Luedi, 2007 |
| CD2 | 1p13 | | X | Mostoslavsky et al., 2001 |
| APOB | 2q24 | | X | Kitsberg et al., 1993 |
| GPD2 | 2q24 | | X | Piras et al., 2000 |
| IGFBP5 | 2q35 | | X | Piras et al., 2000 |
| RPL23 | 2q36 | | X | Greally et al., 1998 |
| COMMD1 | 2p15 | | X | Nabetani et al. (1997) |
| MSX1 | 4p16 | | X | Blin-Wakkach et al., 2001 |
| IL4 | 4p24 | | X | Bix and Locksley, 1998 |
| OSMR | 5p13 | | X | Buettner et al., 2004 |
| PRLR | 5p13 | | X | Buettner et al., 2004 |
| IL7R | 5p13 | | X | Buettner et al., 2004 |
| SEMA5A | 5p15 | | X | Buettner et al., 2004 |
| CDH10 | 5p14 | | X | Buettner et al., 2004 |
| IL13 | 5q23 | | X | Kelly and Locksley, 2000 |
| GABRA6 | 5q34 | | X | Takahashi and Ko, 1993 |
| IGF2R | 6q25 | | X | Kalscheuer et al. (1993); Ogawa et al. (1993b); Killian et al. (2001) |
| SOD2 | 6q25 | | X | Barlow et al., 1991 |
| TCP1 | 6q25 | | X | Barlow et al., 1991 |
| MAS1 | 6q25 | | X | Schweifer et al., 1997; Lyle et al., 2000 |
| PLG | 6q26 | | X | Barlow et al., 1991 |
| EGFR | 7p11 | | X | Wakeling et al. (1998) |
| COBL | 7p12 | | X | Hitchins et al. (2002) |
| DDC | 7p12 | | X | Hitchins et al. (2002) |
| IGFBP1 | 7p13 | | X | Eggermann et al. (1999); Wakeling et al. (2000) |
| COL1A2 | 7q21 | | X | Mizuno et al., 2002 |
| ACTB | 7p22 | | X | Zhang et al., 1994 |
| ACHE | 7q22 | | X | Kitsberg et al., 1993 |
| UBE2H | 7q32 | | X | Yamada et al., 2003 |
| CPA1 | 7q32 | | X | Bentley et al. (2003) |
| HSPC216 | 7q32 | | X | Yamada et al. (2003) |
| CPA2 | 7q32 | | X | Bentley et al. (2003) |
| MKRN1 | 7q34 | | X | Walter and Paulsen, 2003 |
| OR2A1 | 7q35 | | X | Singh et al., 2003 |
| CNTNAP2 | 7q36 | | X | P. P. Luedi, 2007 |
| EN2 | 7q36 | | X | P. P. Luedi, 2007 |
| SDC2 | 8q22 | | X | Buettner et al., 2004 |
| FZD6 | 8q22 | | X | Buettner et al., 2004 |
| NOV | 8q24 | | X | Buettner et al., 2004 |
| NOR1 | 9q22 | | X | P. P. Luedi, 2007 |
| SAMD6 | 9q22 | | X | P. P. Luedi, 2008 |
| DOCK8 | 9p24 | | X | Lerer et al., 2005 |
| C10orf9 | 10p11 | | X | P. P. Luedi, 2007 |
| SFMBT2 | 10p14 | | X | P. P. Luedi, 2007 |
| SFTPD | 10q22 | | X | Lin and Floros, 2002 |
| C10orf28 | 10q24 | | X | P. P. Luedi, 2007 |
| GAS2 | 11p14 | | X | Piras et al., 2000 |
| TPH1 | 11p15 | | X | Buettner et al., 2004 |
| PHEMx | 11p15 | | X | Paulsen et al. (2000); Monk et al. (2006) |
| CD81 | 11p15 | | X | Maecker et al. (1998); Gabriel et al. (1998); Monk et al. (2006) |
| TNNT3 | 11p15 | | X | Yuan et al. (1996) |
| HRAS | 11p15 | | X | Goldberg et al. (2003) |
| CDKN1B | 12p13 | | X | Cost et al. (1997) |
| CD4 | 12p13 | | X | Williamson et al., 1995 |
| YY1 | 14q32 | | X | Yevtodiyenko et al., 2002 |
| WARS | 14q32 | | X | Yevtodiyenko et al., 2002 |
| CYFIP1 | 15q11 | | X | Chai et al. (2003) |
| C15orf2 | 15q11 | | X | Farber et al. (2000) |
| OCA2 | 15q12 | | X | Chai et al. (2003) |
| GABRG3 | 15q12 | | X | Chai et al. (2003) |
| NDNL2 | 15q13 | | X | Chibuk et al., 2001 |
| SLC28A2 | 15q21 | | X | Sandell et al., 2003 |
| SLC30A4 | 15q21 | | X | Sandell et al., 2003 |
| IGFALS | 16p13 | | X | Sano et al., 2001 |
| TP53 | 17p13 | | X | Kitsberg et al., 1993 |
| RPL19 | 17q12 | | X | Piras et al., 2000 |
| ERBB2 | 17q12 | | X | Amiel et al., 1999 |
| OR7A17 | 19p13 | | X | Singh et al., 2003 |
| HRC | 19q13 | | X | Buettner et al., 2004 |
| TH1L | 20q13 | | X | Bonthron et al. (2000) |
| TNFRSF5 | 20q13 | | X | Williamson et al., 1995 |
| KCNB1 | 20q13 | | X | Williamson et al., 1995 |

**Relevant calculated features table**

| **Gene** | **Expression** | **Lenght** | **Band** | **CG content** | **Number of CpGislands** | **size** | **Number of LTR** | **size - count** | **Number of Simple Repeats** | **size** |
| --- | --- | --- | --- | --- | --- | --- | --- | --- | --- | --- |
| TP73 | M | 2234 | 1 | 64.6 | 3 | 752 | 0 | - | 0 | 0 |
| LRRTM1 | P | 2217 | 2 | 58.4 | 2 | 1050 | 1 | 24_7 | 0 | 0 |
| NAP1L5 | P | 1912 | 4 | 42.9 | 0 | 0 | 1 | 12_7 | 2 | 155 |
| PRIM2 | M | 2353 | 6 | 40.7 | 0 | 0 | 0 | - | 1 | 44 |
| PLAGL1 | P | 4354 | 6 | 46.9 | 1 | 317 | 1 | 25 _3 | 1 | 61 |
| HYMAI | P | 5005 | 6 | 42.1 | 1 | 904 | 1 | 13_7 | 2 | 149 |
| PEG10 | P | 6628 | 7 | 44.7 | 2 | 491 | 2 | 42_3 12_4 | 3 | 109 |
| PON1 | P | 2395 | 7 | 41.3 | 0 | 0 | 0 | - | 0 | 0 |
| CALCR | M | 3470 | 7 | 40.4 | 0 | 0 | 0 | - | 0 | 0 |
| PPP1R9A | M | 9705 | 7 | 39.9 | 0 | 0 | 1 | 5_8 | 0 | 0 |
| MEST | P | 2507 | 7 | 45.1 | 1 | 214 | 2 | 42_4 23_3 | 1 | 50 |
| COPG2 | P | 3365 | 7 | 43.1 | 0 | 0 | 0 | - | 1 | 44 |
| CPA4 | M | 2807 | 7 | 48.9 | 1 | 203 | 0 | - | 1 | 59 |
| KLF14 | M | 1383 | 7 | 66.8 | 2 | 889 | 1 | 18_9 | 0 | 0 |
| KCNK9 | M | 1303 | 8 | 60.1 | 2 | 793 | 0 | - | 0 | 0 |
| INPP5F_V2 | P | 4955 | 10 | 43.5 | 1 | 241 | 0 | - | 1 | 24 |
| KCNQ1 | M | 3262 | 11 | 63.4 | 1 | 716 | 1 | 30_4 | 0 | 0 |
| IGF2AS | P | 2056 | 11 | 64 | 1 | 581 | 0 | - | 0 | 0 |
| SMPD1 | M | 2473 | 11 | 59.8 | 1 | 286 | 1 | 6_9 | 0 | 0 |
| IGF2 | P | 1356 | 11 | 63.7 | 3 | 1129 | 1 | 14_18 | 0 | 0 |
| ZNF215 | M | 3658 | 11 | 40.4 | 1 | 240 | 2 | 84_3 84_3 | 0 | 0 |
| H19 | M | 2615 | 11 | 55.9 | 0 | 0 | 2 | 8_10 20_4 | 1 | 35 |
| SLC22A18 | M | 1549 | 11 | 65 | 1 | 342 | 0 | - | 1 | 22 |
| PHLDA2 | M | 937 | 11 | 61.7 | 1 | 597 | 1 | 9_14 | 0 | 0 |
| NDN | P | 1897 | 15 | 52.3 | 2 | 566 | 1 | 57_4 | 0 | 0 |
| MKRN3 | P | 3107 | 15 | 48 | 0 | 0 | 1 | 29_5 | 1 | 123 |
| MAGEL2 | P | 2294 | 15 | 53.7 | 0 | 0 | 2 | 36_7 21_3 | 0 | 0 |
| UBE3A | M | 4491 | 15 | 36.7 | 0 | 0 | 1 | 10_7 | 2 | 59 |
| TCEB3C | M | 1877 | 18 | 64.7 | 2 | 1258 | 0 | - | 1 | 71 |
| NNAT | P | 1338 | 20 | 56.5 | 0 | 0 | 0 | - | 1 | 38 |
| IL4 | B | 921 | 5 | 42.2 | 0 | 0 | 0 | - | 0 | 0 |
| IL13 | B | 1282 | 5 | 52 | 0 | 0 | 0 | - | 0 | 0 |
| OR2A1 | B | 933 | 7 | 56 | 0 | 0 | 0 | - | 0 | 0 |
| SFTPD | B | 1299 | 10 | 57 | 0 | 0 | 1 | 63_5 | 0 | 0 |
| IGFALS | B | 2125 | 16 | 66.3 | 1 | 526 | 1 | 72_17 | 1 | 71 |
| OR7A17 | B | 930 | 19 | 48 | 0 | 0 | 0 | - | 0 | 0 |
| CD2 | B | 1579 | 1 | 43.6 | 0 | 0 | 2 | 29_3 21_3 | 1 | 65 |
| APOB | B | 14121 | 2 | 43.5 | 1 | 207 | 1 | 15_7 | 1 | 46 |
| GPD2 | B | 2796 | 2 | 44 | 0 | 0 | 1 | 68_2 | 0 | 0 |
| IGFBP5 | B | 6316 | 2 | 51.2 | 2 | 469 | 2 | 21_5 22_4 | 5 | 138 |
| RPL23 | B | 594 | 2 | 45 | 0 | 0 | 0 | - | 0 | 0 |
| MSX1 | B | 1944 | 4 | 59.4 | 1 | 523 | 2 | 19_3 6_13 | 1 | 52 |
| OSMR | B | 4171 | 5 | 46 | 1 | 318 | 0 | 0 | 0 | 0 |
| PRLR | B | 3162 | 5 | 44 | 0 | 0 | 0 | 0 | 0 | 0 |
| IL7R | B | 1809 | 5 | 44 | 0 | 0 | 0 | 0 | 0 | 0 |
| SEMA5A | B | 8056 | 5 | 49.9 | 1 | 267 | 0 | 0 | 0 | 0 |
| CDH10 | B | 3261 | 5 | 40.9 | 0 | 0 | 0 | 0 | 0 | 0 |
| GABRA6 | B | 1732 | 5 | 41 | 0 | 0 | 0 | 0 | 0 | 0 |
| SOD2 | B | 1593 | 6 | 46.3 | 0 | 0 | 0 | 0 | 1 | 86 |
| TCP1 | B | 2463 | 6 | 43.2 | 1 | 267 | 0 | 0 | 0 | 0 |
| MAS1 | B | 1135 | 66 | 47 | 0 | 0 | 0 | 0 | 0 | 0 |
| PLG | B | 2732 | 6 | 50 | 0 | 0 | 0 | 0 | 0 | 0 |
| COL1A2 | B | 5411 | 7 | 55 | 0 | 0 | 2 | 9_334 9_12 | 0 | 0 |
| ACTB | B | 1793 | 7 | 55.2 | 0 | 0 | 2 | 10_5 21_7 | 1 | 44 |
| ACHE | B | 2225 | 7 | 65.3 | 1 | 410 | 1 | 5_11 | 2 | 54 |
| UBE2H | B | 2728 | 7 | 46.4 | 1 | 420 | 0 | 0 | 0 | 0 |
| MKRN1 | B | 3116 | 7 | 47.5 | 1 | 320 | 1 | 16_5 | 3 | 175 |
| SDC2 | B | 2485 | 8 | 42.9 | 1 | 371 | 0 | 0 | 0 | 0 |
| FZD6 | B | 3768 | 8 | 38 | 0 | 0 | 0 | 0 | 0 | 0 |
| NOV | B | 2389 | 8 | 45.2 | 1 | 306 | 0 | 0 | 0 | 0 |
| DOCK8 | B | 7257 | 9 | 47.7 | 0 | 0 | 0 | 0 | 1 | 27 |
| GAS2 | B | 2001 | 11 | 37.8 | 0 | 0 | 1 | 14_5 | 0 | 0 |
| TPH1 | B | 1335 | 11 | 41 | 0 | 0 | 0 | 0 | 0 | 0 |
| NGFB | B | 1052 | 1 | 54 | 0 | 0 | 0 | 0 | 0 | 0 |
| COMMD1 | B | 725 | 2 | 50 | 0 | 0 | 0 | 0 | 0 | 0 |
| IGF2R | B | 9090 | 6 | 51.9 | 2 | 496 | 2 | 32_4 16_7 | 1 | 44 |
| EGFR | B | 5616 | 7 | 51.2 | 1 | 309 | 1 | 9_7 | 0 | 0 |
| COBL | B | 5289 | 7 | 53 | 1 | 258 | 0 | 0 | 0 | 0 |
| DDC | B | 1953 | 7 | 49 | 1 | 272 | 0 | 0 | 0 | 0 |
| IGFBP1 | B | 1660 | 7 | 49.8 | 1 | 405 | 1 | 36_3 | 1 | 30 |
| CPA1 | B | 1380 | 7 | 56 | 0 | 0 | 0 | 0 | 0 | 0 |
| HSPC216 | B | 1946 | 7 | 50.6 | 0 | 0 | 0 | 0 | 1 | 32 |
| CPA2 | B | 1306 | 7 | 49 | 0 | 0 | 0 | 0 | 0 | 0 |
| CNTNAP2 | B | 9890 | 7 | 44.8 | 0 | 0 | 0 | 0 | 0 | 0 |
| EN2 | B | 4793 | 7 | 57.9 | 2 | 2384 | 3 | 16_8 50_6 5_8 | 4 | 228 |
| NOR1 | B | 5635 | 9 | 46.9 | 2 | 1227 | 1 | 36_2 | 3 | 171 |
| SAMD6 | B | 7147 | 9 | 54.7 | 1 | 352 | 1 | 99_3 | 1 | 129 |
| C10orf9 | B | 2135 | 10 | 48.9 | 0 | 0 | 0 | 0 | 0 | 0 |
| SFMBT2 | B | 4363 | 10 | 51.4 | 2 | 663 | 0 | 0 | 1 | 27 |
| C10orf28 | B | 3330 | 10 | 43.2 | 0 | 0 | 0 | 0 | 0 | 0 |
| PHEMx | B | 1376 | 11 | 60.4 | 0 | 0 | 1 | 5_7 | 1 | 36 |
| CD81 | B | 1497 | 11 | 62.3 | 1 | 226 | 1 | 8_20 | 1 | 172 |
| TNNT3 | B | 1000 | 11 | 58.3 | 0 | 0 | 1 | 54_4 | 0 | 0 |
| HRAS | B | 1061 | 11 | 62.7 | 1 | 366 | 1 | 12_7 | 2 | 91 |
| CDKN1B | B | 2422 | 12 | 45.5 | 2 | 849 | 0 | 0 | 0 | 0 |
| CYFIP1 | B | 4446 | 15 | 42 | 1 | 423 | 1 | 42_3 | 0 | 0 |
| C15orf2 | B | 3471 | 15 | 55 | 0 | 0 | 0 | 0 | 0 | 0 |
| OCA2 | B | 3136 | 15 | 52 | 1 | 233 | 0 | 0 | 0 | 0 |
| GABRG3 | B | 1536 | 15 | 47 | 0 | 0 | 0 | 0 | 0 | 0 |
| TH1L | B | 2263 | 20 | 51 | 0 | 0 | 0 | 0 | 0 | 0 |
| CD4 | B | 3103 | 12 | 54.5 | 0 | 0 | 0 | 0 | 0 | 0 |
| YY1 | B | 2592 | 14 | 53.5 | 1 | 1072 | 4 | 15_9 90_3 6_24 24_10 | 4 | 420 |
| WARS | B | 2884 | 14 | 49 | 0 | 0 | 0 | 0 | 0 | 0 |
| NDNL2 | B | 1728 | 15 | 48.6 | 1 | 309 | 0 | 0 | 1 | 69 |
| SLC28A2 | B | 2459 | 15 | 50.8 | 1 | 244 | 0 | 0 | 0 | 0 |
| SLC30A4 | B | 4290 | 15 | 42 | 1 | 501 | 1 | 19_3 | 0 | 0 |
| TP53 | B | 2629 | 17 | 53.4 | 0 | 0 | 2 | 15_4 13_3 | 0 | 0 |
| RPL19 | B | 748 | 17 | 52 | 0 | 0 | 0 | 0 | 0 | 0 |
| ERBB2 | B | 4624 | 17 | 60.2 | 0 | 0 | 2 | 24_6 32_4 | 0 | 0 |
| HRC | B | 2430 | 19 | 55.5 | 0 | 0 | 2 | 72_8 63_3 | 4 | 246 |
| TNFRSF5 | B | 1616 | 20 | 52.7 | 0 | 0 | 0 | 0 | 0 | 0 |
| KCNB1 | B | 3756 | 20 | 53.7 | 1 | 582 | 0 | 0 | 0 | 0 |
| GFI1 | P | 2784 | 1 | 56 | 1 | 983 | 1 | 84_5 | 0 | 0 |
| EFNA4 | M | 1276 | 1 | 57.2 | 0 | 0 | 0 | 0 | 1 | 21 |
| HSPA6 | M | 2664 | 1 | 59 | 2 | 1248 | 1 | 10_6 | 0 | 0 |
| BCL2L11 | P | 3422 | 2 | 43.8 | 0 | 0 | 0 | 0 | 1 | 3 |
| HOXD9 | M | 2089 | 2 | 63.1 | 2 | 1332 | 3 | 6_11 5_8 6_8 | 2 | 79 |
| PER2 | M | 6219 | 2 | 51.9 | 0 | 0 | 1 | 18_6 | 0 | 0 |
| PPARG | P | 1883 | 3 | 47.6 | 0 | 0 | 0 | 0 | 1 | 58 |
| POLR2H | M | 821 | 3 | 49 | 0 | 0 | 0 | 0 | 0 | 0 |
| PITX2 | P | 2122 | 4 | 52.3 | 2 | 764 | 0 | 0 | 1 | 25 |
| TLL1 | P | 6654 | 4 | 42 | 2 | 598 | 1 | 40_3 | 1 | 78 |
| NDUFS4 | P | 668 | 5 | 42 | 0 | 0 | 0 | 0 | 0 | 0 |
| ITGB8 | M | 8787 | 7 | 37.9 | 1 | 429 | 2 | 59_2 16_6 | 0 | 0 |
| CDK6 | M | 11611 | 7 | 39.5 | 2 | 797 | 2 | 6_11 6_8 | 6 | 192 |
| PTPRN2 | M | 4767 | 7 | 58.3 | 4 | 964 | 1 | 6_9 | 1 | 44 |
| GADD45G | P | 1078 | 9 | 63 | 1 | 735 | 0 | 0 | 0 | 0 |
| AKR1C2 | P | 1663 | 10 | 45 | 0 | 0 | 1 | 39_3 | 1 | 74 |
| GATA3 | P | 3070 | 10 | 53.7 | 3 | 906 | 2 | 6_14 7_7 | 1 | 49 |
| NRGN | P | 1295 | 11 | 64.6 | 2 | 847 | 0 | 0 | 0 | 0 |
| KLRF1 | P | 1242 | 12 | 35 | 0 | 0 | 0 | 0 | 0 | 0 |
| KLRC3 | P | 1042 | 12 | 39.5 | 0 | 0 | 0 | 0 | 0 | 0 |
| POU4F1 | M | 5015 | 13 | 43.2 | 1 | 258 | 1 | 5_11 | 0 | 0 |
| F10 | M | 1560 | 13 | 58.4 | 1 | 299 | 0 | 0 | 1 | 26 |
| JAG2 | M | 5077 | 14 | 65.9 | 6 | 2458 | 5 | 19_6 6_19 57_7 6_15 41_3 | 2 | 70 |
| SFRS2 | M | 2923 | 17 | 45 | 1 | 729 | 2 | 6_21 18_3 | 1 | 42 |
| GATA6 | M | 3494 | 18 | 56.5 | 2 | 1879 | 2 | 6_5 9_21 | 2 | 63 |
| ELA2 | M | 938 | 19 | 65 | 2 | 721 | 0 | 0 | 0 | 0 |
| ZNF42 | M | 2620 | 19 | 65.3 | 1 | 1241 | 2 | 84_9 84_3 | 0 | 0 |
| SHC1 | M | 1752 | 1 | 60 | 0 | 0 | 0 | 0 | 0 | 0 |
| CYP1B1 | P | 5128 | 2 | 44.3 | 2 | 1155 | 0 | 0 | 0 | 0 |
| SIX3 | P | 1926 | 2 | 61.3 | 2 | 1002 | 3 | 6_19 6_14 6_10 | 3 | 201 |
| OTX1 | M | 2176 | 2 | 60.3 | 2 | 1023 | 2 | 6_14 6_6 | 2 | 83 |
|  |  |  |  |  |  |  |  |  |  |  |

**Pairwise sequence alignment results for the I1 and I2 groups (Wilbur and Lipman, 1983).**

The sequences are presented from its 5’ to its 3’ end, from left to right.

Sequences from I1 group

Sequence_1: CTGCCGAACCACACCTTCCAGGAC

Sequence_2: CGGCGCGCCCGCCGCCTC

Sequence_3: CGCGGCCGCCGCCCCGGGCCCCGCGCCCCC

Sequence_4: CGCTGG

Sequence_5: TCCCCCCCTCTCTC

Sequence_6: CCGCGCCCT

Sequence_7: CCCAGGCCCACAACGCCCCGGGCGCCCCGAAGGCGGTTCCGCCGGCCGCGGCCCCGG

Results of all possible pairwise aligment combinations of I1 group sequences

| Sequence pair | Sequence fragment* | Aligment | Simi-larity Index | Gap Length | Consensus Length |
| --- | --- | --- | --- | --- | --- |
| Sequence_1 *vs* Sequence_2  (1>24) (1>18) | Seq1 (6<1)  Seq2 (1>4) | CGGC  ||||  CGGC | 100 | 0 | 4 |
| Sequence_1 *vs* Sequence_3  (1>24) (1>30) | Seq1 (6<1)  Seq3 (3>6) | CGGC  ||||  CGGC | 100 | 0 | 4 |
| Sequence_1 *vs* Sequence_4  (1>24) (1>6) | Seq1 (1>3)  Seq4 (3>5) | CTG  |||  CTG | 100 | 0 | 3 |
| Sequence_1 *vs* Sequence_5  (1>24) (1>14) | Seq1 (14>16)  Seq5 (7>9) | CCT  |||  CCT | 100 | 0 | 3 |
| Sequence_1 *vs* Sequence_6  (1>24) (1>9) | Seq1 (22<18)  Seq6 (7>9) | CCT  |||  CCT | 100 | 0 | 3 |

| Sequence_1 *vs* Sequence_7  (1>24) (1>57) | Seq1 (6<1)  Seq7 (1>4) | GAAGGTGTGGTTC  ||||| |||||  GAAGGC--GGTTC | 71.4 | 2 | 13 |
| --- | --- | --- | --- | --- | --- |
| Sequence_2 *vs* Sequence_3  (1>18) (1>30) | Seq2 (4>16)  Seq3 (1>13) | CGCGCCCGCCGCC  |||| ||||||||  CGCGGCCGCCGCC | 92.3 | 0 | 13 |
| Sequence_2 *vs* Sequence_4  (1>18) (1>6) | Seq2 (10>12)  Seq4 (1>3) | CGC  |||  CGC | 100 | 0 | 3 |
| Sequence_2 *vs* Sequence_5  (1>18) (1>14) | Seq2 (8>18)  Seq5 (4>14) | CCCGCCGCCTC  ||| | |||  CCCCCTCTCTC | 63.6 | 0 | 11 |
| Sequence_2 *vs* Sequence_6  (1>18) (1>9) | Seq2 (9>17)  Seq6 (1>9) | CCGCCGCCT  |||| |||  CCGCGCGGT | 77.8 | 0 | 9 |
| Sequence_2 *vs* Sequence_7  (1>18) (1>57) | Seq2 (17<1)  Seq7 (4>20) | AGGCGGCGGGCGCGCCG  |||| ||| |||  AGGCCCACAACGCCCCG | 58.8 | 0 | 17 |
| Sequence_3 *vs* Sequence_4  (1>30) (1>6) | Seq3 (22>24)  Seq4 (1>3) | CGC  ||||  CGC | 100 | 0 | 3 |
| Sequence_3 *vs* Sequence_5  (1>30) (1>14) | Seq3 (16<14)  Seq5 (5>7) | CCC  |||  CCC | 100 | 0 | 3 |
| Sequence_3 *vs* Sequence_6  (1>30) (1>9) | Seq3 (6<1)  Seq6 (1>4) | CCGCGCCC  ||||||||  CCGCGCCC | 100 | 0 | 8 |
| Sequence_3 *vs* Sequence_7  (1>30) (1>57) | Seq3 (19<2)  Seq7 (15>42) | GCCCGGGGCG--------GCGG--CCGC  |||| ||||| |||| ||||  GCCCCGGGCGCCCCGAAGGCGGTTCCGC | 56.7 | 10 | 28 |
| Sequence_4 *vs* Sequence_5  (1>24) (1>18) |  | No aligment | - | - | - |
| Sequence_4 *vs* Sequence_6  (1>6) (1>9) | Seq4 (3<1)  Seq6 (3>5) | GGC  |||  GGC | 100 | 0 | 3 |

| Sequence_4 *vs* Sequence_7  (1>6) (1>57) | Seq4 (6<1)  Seq7 (2>5) | CCAG  ||||  CCAG | 100 | 0 | 4 |
| --- | --- | --- | --- | --- | --- |
| Sequence_5 *vs* Sequence_6  (1>14) (1>9) | Seq5 (6>9)  Seq6 (1>4) | CCCT  ||||  CCCT | 100 | 0 | 4 |
| Sequence_5 *vs* Sequence_7  (1>14) (1>57) | Seq5 (5>7)  Seq7 (7>9) | CCC  |||  CCC | 100 | 0 | 3 |
| Sequence_6 *vs* Sequence_7  (1>9) (1>57) | Seq6 (3>8)  Seq7 (22>27) | GCGCCC  ||||||  GCGCCC | 100 | 0 | 6 |

*: Numbers denote the sequence positions of each sequences involved in a given pairwise aligment, that limited the extremes of the sequence fragments with a similarity level good enough to be aligned. “>” denotes that sequence fragment is into the complementary sequence.

Sequences from I2 group

Sequence_1: GGAGGAGGAGGA

Sequence_2: ATCTTACAAAAAAAAAAAAAAAAAA

Sequence_3: TATATATATATAA

Sequence_4: AGAAGCTCTCAGAGGAGAACAACAACCTTCGAGAGCAGGTGG

Sequence_5: CCGCCGCCTCCA

Sequence_6: TTTTC

Sequence_7: GGCGGCTGCGGCTGCCGCGCCCGGTGCTGCCCAGCGCTGCGG

Sequence_8: CAAAAAAAAAAAAAAAAAAAAAA

Sequence_9: TATTCGACATCAAAAAATTCATACTGAAGCGAAGGCCTATAAATGCAATAAATGTGGGAAAGCCTTCAGCCGAAGTGCAGACCT

Sequence_10: AAAACTGCATACTGGAGATAAGTCCTGAAAATGTAAAAAATGTAGGAAAACCTTCAACCGGAGTTCAGAACTTATTTAACATCA

Sequence_11: GGGGGGGA

Sequence_12: CTTTTTCTTCTTCCTCCTTT

Sequence_13: TTAAAAATTATATATATAAGAATATAAAA

Sequence_14: CGGGCCCTGAGTGTCTGGGAGGGCCCAAGCACCTCC

Sequence_15: GGCCTCCTCAAAAGAGCGCAG

Sequence_16: AAAACAAAAA

| Sequence pair | Sequence fragment* | Aligment | Simi-larity Index | Gap Length | Consensus Length |
| --- | --- | --- | --- | --- | --- |
| Sequence_1 *vs* Sequence_2 |  | No aligment |  |  |  |
| Sequence_1 *vs* Sequence_3 |  | No aligment |  |  |  |
| Sequence_1 *vs* Sequence_4  (1>12) (1>42) | Seq1 (7>10)  Seq4 (14>17) | GGAG  ||||  GGAG | 100 | 0 | 4 |
| Sequence_1 *vs* Sequence_5  (1>12) (1>12) | Seq1 (5<1)  Seq5 (7>11) | CCTCC  |||||  CCTCC | 100 | 0 | 5 |
| Sequence_1 *vs* Sequence_6 |  | No aligment |  |  |  |
| Sequence_1 *vs* Sequence_7 |  | No aligment |  |  |  |
| Sequence_1 *vs* Sequence_8 |  | No aligment |  |  |  |
| Sequence_1 *vs* Sequence_9  (1>12) (1>84) | Seq1 (8<4)  Seq9 (63>65) | CCT  |||  CCT | 100 | 0 | 3 |
| Sequence_1 *vs* Sequence_10  (1>12) (1>84) | Seq1 (9>12)  Seq10 (44>47) | AGGA  ||||  AGGA | 100 | 0 | 4 |
| Sequence_1 *vs* Sequence_11  (1>12) (1>8) | Seq1 (7>9)  Seq11 (6>8) | GGA  |||  GGA | 100 | 0 | 3 |
| Sequence_1 *vs* Sequence_12  (1>12) (1>20) | Seq1 (9<1)  Seq12 (12>18) | TCCTCCT  |||||||  TCCTCCT | 100 | 0 | 7 |
| Sequence_1 *vs* Sequence_13 |  | No aligment |  |  |  |
| Sequence_1 *vs* Sequence_14  (1>12) (1>36) | Seq1 (5<1)  Seq14 (32>36) | CCTCC  |||||  CCTCC | 100 | 0 | 5 |
| Sequence_1 *vs* Sequence_15  (1>12) (1>21) | Seq1 (4<1)  Seq15 (3>6) | CCTC  ||||  CCTC | 100 | 0 | 4 |
| Sequence_1 *vs* Sequence_16 |  | No aligment |  |  |  |
| Sequence_2 *vs* Sequence_3  (1>25) (1>13) | Seq2 (6<4)  Seq3 (11>13) | TAA  |||  TAA | 100 | 0 | 3 |
| Sequence_2 *vs* Sequence_4  (1>25) (1>42) | Seq2 (5<3)  Seq4 (3>5) | AAG  |||  AAG | 100 | 0 | 3 |
| Sequence_2 *vs* Sequence_5 |  | No aligment |  |  |  |
| Sequence_2 *vs* Sequence_6  (1>25) (1>5) | Seq2 (11<6)  Seq6 (1>4) | TTTT  ||||  TTTT | 100 | 0 | 4 |
| Sequence_2 *vs* Sequence_7 |  | No aligment |  |  |  |
| Sequence_2 *vs* Sequence_8  (1>25) (1>23) | Seq2 (7>25)  Seq8 (1>19) | CAAAAAAAAAAAAAAAAAA  |||||||||||||||||||  CAAAAAAAAAAAAAAAAAA | 100 | 0 | 19 |
| Sequence_2 *vs* Sequence_9  (1>25) (1>84) | Seq2 (9<1)  Seq9 (12>18) | ACAAAAAAAAA  ||| ||||||  ACATTAAAAAA | 81.8 | 0 | 11 |
| Sequence_2 *vs* Sequence_10  (1>25) (1>84) | Seq2 (9>25)  Seq10 (28>50) | AAAAAAAAAAAAA------AAAA  |||| ||||||| ||||  AAAATGTAAAAAATGTAGGAAAA | 58.3 | 6 | 23 |
| Sequence_2 *vs* Sequence_11 |  | No aligment |  |  |  |
| Sequence_2 *vs* Sequence_12  (1>25) (1>20) | Seq2 (8>25)  Seq12 (20<1) | AAAGGAGGAAGAAGAAAA  ||| | || || ||||  AAAAAAAAAAAAAAAAAA | 66.7 | 0 | 18 |
| Sequence_2 *vs* Sequence_13  (1>25) (1>29) | Seq2 (9<1)  Seq13 (12>18) | TTATATATATAAGAATATAAAA  ||| | | | || || | ||||  TTACAAAAAAAAAAAAAAAAAA | 68.2 | 0 | 22 |
| Sequence_2 *vs* Sequence_14  (1>25) (1>36) | Seq2 (3>5)  Seq14 (29<25) | CTT  |||  CTT | 100 | 0 | 3 |
| Sequence_2 *vs* Sequence_15  (1>25) (1>21) | Seq2 (7>11)  Seq15 (9>13) | CAAAA  |||||  CAAAA | 100 | 0 | 5 |
| Sequence_2 *vs* Sequence_16  (1>25) (1>10) | Seq2 (16>25)  Seq16 (1>10) | AAAAAAAAAA  |||| |||||  AAAACAAAAA | 90 | 0 | 10 |
| Sequence_3 *vs* Sequence_4 |  | No aligment |  |  |  |
| Sequence_3 *vs* Sequence_5 |  | No aligment |  |  |  |
| Sequence_3 *vs* Sequence_6 |  | No aligment |  |  |  |
| Sequence_3 *vs* Sequence_7 |  | No aligment |  |  |  |
| Sequence_3 *vs* Sequence_8 |  | No aligment |  |  |  |
| Sequence_3 *vs* Sequence_9  (1>13) (1>84) | Seq3 (8>10)  Seq9 (21>23) | ATA  |||  ATA | 100 | 0 | 3 |
| Sequence_3 *vs* Sequence_10  (1>13) (1>84) | Seq3 (7>13)  Seq10 (73>79) | TATATAA  ||| |||  TATTTAA | 85.7 | 0 | 7 |
| Sequence_3 *vs* Sequence_11 |  | No aligment |  |  |  |
| Sequence_3 *vs* Sequence_12 |  | No aligment |  |  |  |
| Sequence_3 *vs* Sequence_13  (1>13) (1>29) | Seq3 (3>13)  Seq13 (9>19) | TATATATATAA  |||||||||||  TATATATATAA | 100 | 0 | 11 |
| Sequence_3 *vs* Sequence_14 |  | No aligment |  |  |  |
| Sequence_3 *vs* Sequence_15 |  | No aligment |  |  |  |
| Sequence_3 *vs* Sequence_16 |  | No aligment |  |  |  |
| Sequence_4 *vs* Sequence_5  (1>42) (1>12) | Seq4 (6>8)  Seq5 (8>10) | CTC  |||  CTC | 100 | 0 | 3 |
| Sequence_4 *vs* Sequence_6  (1>42) (1>5) | Seq4 (3>13)  Seq6 (9>19) | TTC  |||  TTC | 100 | 0 | 3 |
| Sequence_4 *vs* Sequence_7  (1>42) (1>42) | Seq4 (3>13)  Seq7 (9>19) | GCTCTCAGAGGAGAACAACAACCTTCGAGAGCAGGT  ||| | |||  GCTGCCGCGCCC---------------------GGT | 21.1 | 21 | 36 |
| Sequence_4 *vs* Sequence_8  (1>42) (1>23) | Seq3 (21>23)  Seq13 (22>24) | AAC  |||  AAC | 100 | 0 | 3 |
| Sequence_4 *vs* Sequence_9  (1>42) (1>84) | Seq4 (3>41)  Seq9 (73<22) | AAGCTCTCAGAGGAGAACAACAACCTTCGAGA-GCAG  |||| |||| ||| ||||  AAGCCTTCAGC----------------CGAAGTGCAG | 38.5 | 17 | 37 |
| Sequence_4 *vs* Sequence_10  (1>42) (1>84) | Seq4 (3>39)  Seq10 (9>19) | AAGTTCTGAACTCCGGTTGAAGGTTTTCCTACATTTTTTACATTTTCAGG  ||| ||| | | | ||| ||||  AAGCTCTCAGAGGAGAACAACAACCTTCGAGAG-------------CAGG | 31.4 | 13 | 50 |
| Sequence_4 *vs* Sequence_11  (1>42) (1>8) | Seq4 (41>43)  Seq11 (6>8) | GGA  |||  GGA | 100 | 0 | 3 |
| Sequence_4 *vs* Sequence_12  (1>42) (1>20) | Seq4 (3>13)  Seq12 (9>19) | AGG  |||  AGG | 100 | 0 | 3 |
| Sequence_4 *vs* Sequence_13  (1>42) (1>29) | Seq4 (3>13)  Seq13 (9>19) | AAG  |||  AAG | 100 | 0 | 3 |
| Sequence_4 *vs* Sequence_14  (1>42) (1>36) | Seq4 (7>28)  Seq14 (14>34) | TCTCAGAGGAGAACAACAACCT  ||| |||| ||| ||||  TCTGGGAGGGCC-CAAGCACCT | 60.90 | 1 | 22 |
| Sequence_4 *vs* Sequence_15  (1>42) (1>21) | Seq4 (5>17)  Seq15 (17<2) | GCTCTCA-GAGGAG  ||||| ||||||  GCTCTTTTGAGGAG | 73.3 | 1 | 14 |
| Sequence_4 *vs* Sequence_16 |  | No aligment |  |  |  |
| Sequence_5 *vs* Sequence_6 |  | No aligment |  |  |  |
| Sequence_5 *vs* Sequence_7  (1>12) (1>42) | Seq5 (8<1)  Seq7 (4>11) | GGCGGCGG  ||| ||||  GGCTGCGG | 87.5 | 0 | 8 |
| Sequence_5 *vs* Sequence_8 |  | No aligment |  |  |  |
| Sequence_5 *vs* Sequence_9  (1>12) (1>84) | Seq5 (8<1)  Seq9 (4>11) | GCCT  ||||  GCCT | 100 | 0 | 4 |
| Sequence_5 *vs* Sequence_10  (1>12) (1>84) | Seq5 (8<1)  Seq10 (4>11) | CTCCA  |||||  CTCCA | 100 | 0 | 5 |
| Sequence_5 *vs* Sequence_11 |  | No aligment |  |  |  |
| Sequence_5 *vs* Sequence_12  (1>12) (1>20) | Seq5 (7>10)  Seq12 (13>16) | CCTC  ||||  CCTC | 100 | 0 | 4 |
| Sequence_5 *vs* Sequence_13 |  | No aligment |  |  |  |
| Sequence_5 *vs* Sequence_14  (1>12) (1>36) | Seq5 (7>11)  Seq14 (22<16) | CCTCC  |||||  CCTCC | 100 | 0 | 5 |
| Sequence_5 *vs* Sequence_15  (1>12) (1>21) | Seq5 (3>10)  Seq15 (2>9) | GCCGCCTC  ||| ||||  GCCTCCTC | 87.5 | 0 | 8 |
| Sequence_5 *vs* Sequence_16 |  | No aligment |  |  |  |
| Sequence_6 *vs* Sequence_7 |  | No aligment |  |  |  |
| Sequence_6 *vs* Sequence_8  (1>5) (1>23) | Seq6 (4<1)  Seq8 (15>18) | AAAA  ||||  AAAA | 100 | 0 | 4 |
| Sequence_6 *vs* Sequence_9  (1>5) (1>84) | Seq6 (3>5)  Seq9 (15>18) | TTC  |||  TTC | 100 | 0 | 3 |
| Sequence_6 *vs* Sequence_10  (1>5) (1>84) | Seq6 (3>5)  Seq10 (53>55) | TTC  |||  TTC | 100 | 0 | 3 |
| Sequence_6 *vs* Sequence_11 |  | No aligment |  |  |  |
| Sequence_6 *vs* Sequence_12  (1>5) (1>20) | Seq6 (1>5)  Seq12 (3>7) | TTTTC  |||||  TTTTC | 100 | 0 | 5 |
| Sequence_6 *vs* Sequence_13  (1>5) (1>20) | Seq6 (1>4)  Seq13 (6<1) | TTTT  ||||  TTTT | 100 | 0 | 4 |
| Sequence_6 *vs* Sequence_14  (1>5) (1>36) | Seq6 (4>6)  Seq14 (14>16) | TCT  |||  TCT | 100 | 0 | 3 |
| Sequence_6 *vs* Sequence_15  (1>5) (1>21) | Seq6 (1>4)  Seq15 (13<8) | TTTT  ||||  TTTT | 100 | 0 | 4 |
| Sequence_6 *vs* Sequence_16  (1>5) (1>10) | Seq6 (1>4)  Seq16 (4<1) | TTTT  ||||  TTTT | 100 | 0 | 4 |
| Sequence_7 *vs* Sequence_8 |  | No aligment |  |  |  |
| Sequence_7 *vs* Sequence_9  (1>42) (1>84) | Seq7 (38<3)  Seq9 (28>72) | AGCGCTGG---------GCAGCACCGGGCGCGGCAGCCGCAGCCG  |||| || ||| | | | | ||||||  AGCGAAGGCCTATAAATGCAATAAATGTGGGAAAGCCTTCAGCCG | 41.3 | 9 | 45 |
| Sequence_7 *vs* Sequence_10  (1>42) (1>84) | Seq7 (9>11)  Seq16 (58<60) | CGG  |||  CGG | 100 | 0 | 3 |
| Sequence_7 *vs* Sequence_11 |  | No aligment |  |  |  |
| Sequence_7 *vs* Sequence_12 |  | No aligment |  |  |  |
| Sequence_7 *vs* Sequence_13 |  | No aligment |  |  |  |
| Sequence_7 *vs* Sequence_14  (1>42) (1>36) | Seq7 (23>34)  Seq14 (33<15) | GGTGCTG-------CCCAG  |||||| |||||  GGTGCTTGGGCCCTCCCAG | 55 | 7 | 19 |
| Sequence_7 *vs* Sequence_15  (1>42) (1>21) | Seq7 (5>21)  Seq15 (17<1) | GCTGCGGCTGCCGCGCC  ||| |||  GCTCTTTTGAGGAGGCC | 35.3 | 0 | 17 |
| Sequence_7 *vs* Sequence_16 |  | No aligment |  |  |  |
| Sequence_8 *vs* Sequence_9  (1>23) (1>84) | Seq8 (1>7)  Seq9 (11>17) | CAAAAAA  |||||||  CAAAAAA | 100 | 0 | 7 |
| Sequence_8 *vs* Sequence_10  (1>23) (1>84) | Seq8 (2>23)  Seq10 (29<50) | AAAAAAAAAAAAAAAAAAAAAA  ||| |||||| | ||||  AAATGTAAAAAATGTAGGAAAA | 63.6 | 0 | 22 |
| Sequence_8 *vs* Sequence_11 |  | No aligment |  |  |  |
| Sequence_8 *vs* Sequence_12  (1>23) (1>84) | Seq8 (5>23)  Seq12 (20<1) | AAAAAAAAAAAAAAAAAAA  ||| | || || |||||  AAAGGAGGAAGAAGAAAAA | 68.4 | 0 | 19 |
| Sequence_8 *vs* Sequence_13  (1>23) (1>29) | Seq8 (15>25)  Seq13 (3>31) | AAAAA------------------AAAACA  ||||| ||||  AAAAATTATATATATAAGAATATAAAATT | 30 | 18 | 29 |
| Sequence_8 *vs* Sequence_14  (1>23) (1>36) | Seq8 (1>3)  Seq14 (26>29) | CAA  |||  CAA | 100 | 0 | 3 |
| Sequence_8 *vs* Sequence_15  (1>23) (1>21) | Seq8 (1>5)  Seq15 (9>13) | CAAAA  |||||  CAAAA | 100 | 0 | 5 |
| Sequence_8 *vs* Sequence_16  (1>23) (1>10) | Seq8 (12>21)  Seq16 (1>10) | AAAAAAAAAA  |||| |||||  AAAACAAAAA | 90 | 0 | 10 |
| Sequence_9 *vs* Sequence_10  (1>84) (1>84) | Seq9 (41>81)  Seq10 (29>69) | AAATGCAATAAATGTGGGAAAGCCTTCAGCCGAAGTGCAGA  ||||| || |||||| ||||| |||||| ||| ||| ||||  AAATGTAAAAAATGTAGGAAAACCTTCAACCGGAGTTCAGA | 82.9 | 0 | 41 |
| Sequence_9 *vs* Sequence_11  (1>84) (1>8) | Seq9 (56>59)  Seq11 (5>8) | GGGA  ||||  GGGA | 100 | 0 | 4 |
| Sequence_9 *vs* Sequence_12  (1>84) (1>20) | Seq9 (15>34)  Seq12 (20>1) | AAATTCATACTGAAGCGAAG  ||| | | |||  AAAGGAGGAAGAAGAAAAAG | 40 | 0 | 20 |
| Sequence_9 *vs* Sequence_13  (1>84) (1>29) | Seq9 (1>5)  Seq13 (24<18) | TATTC  |||||  TATTC | 100 | 0 | 5 |
| Sequence_9 *vs* Sequence_14  (1>84) (1>36) | Seq9 (6>9  Seq14 (15<10) | GACA  ||||  GACA | 100 | 0 | 4 |
| Sequence_9 *vs* Sequence_15  (1>84) (1>21) | Seq9 (10>15)  Seq15 (8>13) | TCAAAA  ||||||  TCAAAA | 100 | 0 | 6 |
| Sequence_9 *vs* Sequence_16  (1>84) (1>10) | Seq9 (11>16)  Seq16 (5>10) | CAAAAA  ||||||  CAAAAA | 100 | 0 | 6 |
| Sequence_10 *vs* Sequence_11  (1>84) (1>8) | Seq10 (14>16)  Seq11 (6>8) | GGA  |||  GGA | 100 | 0 | 3 |
| Sequence_10 *vs* Sequence_12  (1>84) (1>20) | Seq10 (51>54)  Seq12 (16>19) | CCTT  ||||  CCTT | 100 | 0 | 4 |
| Sequence_10 *vs* Sequence_13  (1>84) (1>29) | Seq10 (1>38)  Seq13 (4>29) | AAAACTGCATACTGGAGATAAGTCCTGAAAATGTAAAA  |||| | ||| ||||| ||| |||||  AAAATTATATAT-----ATAAG-------AATATAAAA | 52.5 | 12 | 38 |
| Sequence_10 *vs* Sequence_14  (1>84) (1>36) | Seq10 (24>58)  Seq14 (6>29) | CCTGAAAATGTAAAAAATGTAGGAAAACCTTCAAC  ||||| ||| | | |||  CCTGAG-----------TGTCTGGGAGGGCCCAAG | 36.1 | 11 | 35 |
| Sequence_10 *vs* Sequence_15  (1>84) (1>21) | Seq10 (47<3)  Seq15 (5>21) | TCCTACATTTTTTACATTTTCAGGACTTATCTCCAGTATGCAG  |||| | ||  TCCTCAAAAGAGC--------------------------GCAG | 20.5 | 26 | 43 |
| Sequence_10 *vs* Sequence_16  (1>84) (1>10) | Seq10 (29>38)  Seq16 (1>10) | AAATCTAAAA  ||| ||||  AAAACAAAAA | 70 | 0 | 10 |
| Sequence_11 *vs* Sequence_12 |  | No aligment |  |  |  |
| Sequence_11 *vs* Sequence_13 |  | No aligment |  |  |  |
| Sequence_11 *vs* Sequence_14  (1>8) (1>36) | Seq11 (8<6)  Seq14 (34>36) | TCC  |||  TCC | 100 | 0 | 3 |
| Sequence_11 *vs* Sequence_15 |  | No aligment |  |  |  |
| Sequence_11 *vs* Sequence_16 |  | No aligment |  |  |  |
| Sequence_12 *vs* Sequence_13  (1>20) (1>29) | Seq12 (20<1)  Seq13 (5>30) | AAAGGAGG-----AAGAAGAAAAAGA  ||| | ||||| | |||  AAATTATATATATAAGAATATAAAAT | 48.1 | 5 | 26 |
| Sequence_12 *vs* Sequence_14  (1>20) (1>36) | Seq12 (17<11)  Seq14 (18>22) | GGAGG  |||||  GGAGG | 100 | 0 | 5 |
| Sequence_12 *vs* Sequence_15  (1>20) (1>21) | Seq12 (17<11)  Seq15 (18>22) | CCTCCT  ||||||  CCTCCT | 100 | 0 | 6 |
| Sequence_12 *vs* Sequence_16  (1>20) (1>10) | Seq12 (20<2)  Seq16 (2>10) | AAAGGAGGAAGAAGAAAAA  ||| |||||  AAAC----------AAAAA | 40 | 10 | 19 |
| Sequence_13 *vs* Sequence_14  (1>29) (1>36) | Seq13 (18>20)  Seq14 (27>29) | AAG  |||  AAG | 100 | 0 | 3 |
| Sequence_13 *vs* Sequence_15  (1>29) (1>21) | Seq13 (26>29)  Seq15 (10>13) | AAAA  ||||  AAAA | 100 | 0 | 4 |
| Sequence_13 *vs* Sequence_16  (1>29) (1>10) | Seq13 (3>7)  Seq16 (6>10) | AAAAA  |||||  AAAAA | 100 | 0 | 5 |
| Sequence_14 *vs* Sequence_15  (1>36) (1>21) | Seq14 (22<5)  Seq15 (6>23) | CCTCCCAGACACTCAGGG  |||| | | |||  CCTCAAAAGAGCGCAGGG | 61.1 | 0 | 18 |
| Sequence_14 *vs* Sequence_16  (1>36) (1>10) | Seq14 (26>28)  Seq16 (5>7) | CAA  |||  CAA | 100 | 0 | 3 |
| Sequence_15 *vs* Sequence_16  (1>21) (1>10) | Seq15 (9>13)  Seq16 (5>9) | CAAAA  |||||  CAAAA | 100 | 0 | 5 |

*: Numbers denote the sequence positions of each sequences involved in a given pairwise aligment, that limited the extremes of the sequence fragments with a similarity level good enough to be aligned. “>” denotes that sequence fragment is into the complementary sequence.

**References**

Kaghad, M., Bonnet, H., Yang, A., Creancier, L., Biscan, J. C., Valent, A., Minty, A., Chalon, P., Lelias, J. M., Dumont, X., Ferrara, P., McKeon, F., and Caput, D. 1997. Monoallelically expressed gene related to p53 at 1p36, a region frequently deleted in neuroblastoma and other human cancers. *Cell* **90**:809–819.

Mai, M., Qian, C., Yokomizo, A., Tindall, D. J., Bostwick, D., Polychronakos, C., Smith, D. I., and Liu,W. 1998. Loss of imprinting and allele switching of p73 in renal cell carcinoma. *Oncogene* **17**:1739–1741.

Cai, Y. C., Yang, G. Y., Nie, Y.,Wang, L. D., Zhao, X., Song, Y. L., Seril, D. N., Liao, J., Xing, E. P., and Yang, C. S. 2000. Molecular alterations of p73 in human esophageal squamous cell carcinomas: loss of heterozygosity occurs frequently; loss of imprinting and elevation of p73 expression may be related to defective p53. *Carcinogenesis* **21**:683–689.

Francks C, Maegawa S, Laurén J, Abrahams BS, Velayos-Baeza A, Medland SE, Colella S, Groszer M, McAuley EZ, Caffrey TM, Timmusk T, Pruunsild P, Koppel I, Lind PA, Matsumoto-Itaba N, Nicod J, Xiong L, Joober R, Enard W, Krinsky B, Nanba E, Richardson AJ, Riley BP, Martin NG, Strittmatter SM, Möller HJ, Rujescu D, St Clair D, Muglia P, Roos JL, Fisher SE, Wade-Martins R, Rouleau GA, Stein JF, Karayiorgou M, Geschwind DH, Ragoussis J, Kendler KS, Airaksinen MS, Oshimura M, DeLisi LE, Monaco AP. LRRTM1 on chromosome 2p12 is a maternally suppressed gene that is associated paternally with handedness and schizophrenia. *Mol Psychiatry*. 2007 Dec;**12**:1129-39, 1057

Wood AJ, Roberts RG, Monk D, Moore GE, Schulz R, Oakey RJ. A screen for retrotransposed imprinted genes reveals an association between X chromosome homology and maternal germ-line methylation. *PLoS Genet*. 2007 Feb 9;**3**(2):e20

Smith RJ, Dean W, Konfortova G, Kelsey G. Identification of novel imprinted genes in a genome-wide screen for maternal methylation. *Genome Res*. 2003 Apr;**13**(4):558-69.

Pant PV, Tao H, Beilharz EJ, Ballinger DG, Cox DR, Frazer KA. Analysis of allelic differential expression in human white blood cells. *Genome Res*. 2006 Mar;**16**(3):331-9

Kamiya, M., Judson, H., Okazaki, Y., Kusakabe, M., Muramatsu, M., Takada, S., Takagi, N., Arima, T., Wake, N., Kamimura, K., Satomura, K., Hermann, R., Bonthron, D. T., and Hayashizaki, Y. 2000. The cell cycle control gene ZAC/PLAGL1 is imprinted–a strong candidate gene for transient neonatal diabetes. *Hum. Mol. Genet*. **9**:453–460.

Arima, T., Drewell, R. A., Oshimura, M., Wake, N., and Surani, M. A. 2000. A novel imprinted gene, HYMAI, is located within an imprinted domain on human chromosome 6 containing ZAC. *Genomics* **67**:248–255.

Ono, R., Kobayashi, S., Wagatsuma, H., Aisaka, K., Kohda, T., Kaneko-Ishino, T., and Ishino, F. 2001. A retrotransposon-derived gene, PEG10, is a novel imprinted gene located on human chromosome 7q21. *Genomics* **73**:232–237.

Okita C, Meguro M, Hoshiya H, Haruta M, Sakamoto YK, Oshimura M. A new imprinted cluster on the human chromosome 7q21-q31, identified by human-mouse monochromosomal hybrids. *Genomics*. 2003 Jun;**81**(6):556-9

Nakabayashi, K., Makino, S., Minagawa, S., Smith, A. C., Bamforth, J. S., Stanier, P., Preece, M., Parker-Katiraee, L., Paton, T., Oshimura, M., Mill, P., Yoshikawa, Y., Hui, C. C., Monk, D., Moore, G. E., and Scherer, S. W. 2004. Genomic imprinting of PPP1R9A encoding neurabin I in skeletal muscle and extra-embryonic tissues. *J. Med. Genet.* **41**:601–608

Kobayashi, S., Kohda, T., Miyoshi, N., Kuroiwa, Y., Aisaka, K., Tsutsumi, O., Kaneko-Ishino, T., and Ishino, F. 1997. Human PEG1/MEST, an imprinted gene on chromosome 7. *Hum. Mol. Genet.* **6**:781–786

Yamasaki K, Hayashida S, Miura K, Masuzaki H, Ishimaru T, Niikawa N, Kishino T. The novel gene, gamma2-COP (COPG2), in the 7q32 imprinted domain escapes genomic imprinting. *Genomics*. 2000 Sep 15;**68**(3):330-5.

Kayashima, T., Yamasaki, K., Yamada, T., Sakai, H., Miwa, N., Ohta, T., Yoshiura, K., Matsumoto, N., Nakane, Y., Kanetake, H., Ishino, F., Niikawa, N., and Kishino, T. 2003. The novel imprinted carboxypeptidase A4 gene (CPA4) in the 7q32 imprinting domain. *Hum. Genet.* **112**:220–226.

Parker-Katiraee L, Carson AR, Yamada T, Arnaud P, Feil R, Abu-Amero SN, Moore GE, Kaneda M, Perry GH, Stone AC, Lee C, Meguro-Horike M, Sasaki H, Kobayashi K, Nakabayashi K, Scherer SW. Identification of the imprinted KLF14 transcription factor undergoing human-specific accelerated evolution. *PLoS Genet*. 2007 May 4;**3**(5):e65.

Ruf N, Bähring S, Galetzka D, Pliushch G, Luft FC, Nürnberg P, Haaf T, Kelsey G, Zechner U. Sequence-based bioinformatic prediction and QUASEP identify genomic imprinting of the KCNK9 potassium channel gene in mouse and human. *Hum Mol Genet*. 2007 Nov 1;**16**(21):2591-9

Lee, M. P., Hu, R. J., Johnson, L. A., and Feinberg, A. P. 1997. Human KVLQT1 gene shows tissue-specific imprinting and encompasses Beckwith-Wiedemann syndrome chromosomal rearrangements. *Nat. Genet.* **15**:181–185

Okutsu, T., Kuroiwa, Y., Kagitani, F., Kai, M., Aisaka, K., Tsutsumi, O., Kaneko, Y., Yokomori, K., Surani, M. A., Kohda, T., Kaneko-Ishino, T., and Ishino, F. 2000. Expression and imprinting status of human PEG8/IGF2AS, a paternally expressed antisense transcript from the IGF2 locus, in Wilms’ tumors. *J. Biochem.* **127**:475–483

Simonaro, C. M., Park, J. H., Eliyahu, E., Shtraizent, N., McGovern, M. M., and Schuchman, E. H. 2006. Imprinting at the SMPD1 Locus: Implications for Acid Sphingomyelinase- Deficient Niemann-Pick Disease. *Am. J. Hum. Genet.* **78**:865–870.

Ogawa, O., Eccles, M. R., Szeto, J., McNoe, L. A., Yun, K., Maw, M. A., Smith, P. J., and Reeve, A. E. 1993*a*. Relaxation of insulin-like growth factor II gene imprinting implicated in Wilms’ tumour. *Nature* **362**:749–751.

Ogawa, O., McNoe, L. A., Eccles, M. R., Morison, I. M., and Reeve, A. E. 1993*b*. Human insulin-like growth factor type I and type II receptors are not imprinted. *Hum. Mol. Genet.* **2**:2163–2165.

Ohlsson, R., Nystrom, A., Pfeifer-Ohlsson, S., Tohonen, V., Hedborg, F., Schofield, P., Flam, F., and Ekstrom, T. J. 1993. IGF2 is parentally imprinted during human embryogenesis and in the Beckwith-Wiedemann syndrome. *Nat. Genet.* **4**:94–97.

Rainier, S., Johnson, L. A., Dobry, C. J., Ping, A. J., Grundy, P. E., and Feinberg, A. P. 1993. Relaxation of imprinted genes in human cancer. *Nature* **362**:747–749.

Alders, M., Ryan, A., Hodges, M., Bliek, J., Feinberg, A. P., Privitera, O., Westerveld, A., Little, P. F., and Mannens, M. 2000. Disruption of a novel imprinted zinc-finger gene, ZNF215, in Beckwith-Wiedemann syndrome. *Am. J. Hum. Genet.* **66**:1473–1484.

Zhang, Y. and Tycko, B. 1992. Monoallelic expression of the human H19 gene. *Nat. Genet.* **1**:40–44.

Cooper, P. R., Smilinich, N. J., Day, C. D., Nowak, N. J., Reid, L. H., Pearsall, R. S., Reece, M., Prawitt, D., Landers, J., Housman, D. E., Winterpacht, B. B. R. C. A., Zabel, B. U., Pelletier, J., Weissman, B. E., Shows, T. B., and Higgins, M. J. 1998. Divergently transcribed overlapping genes expressed in liver and kidney and located in the 11p15.5 imprinted domain. *Genomics* **49**:38–51.

Bajaj, V., Markandaya, M., Krishna, L., and Kumar, A. 2004. Paternal imprinting of the SLC22A1LS gene located in the human chromosome segment 11p15.5. *BMC Genet.* **5**:13.

Qian, N., Frank, D., O’Keefe, D., Dao, D., Zhao, L., Yuan, L., Wang, Q., Keating, M., Walsh, C., and Tycko, B. 1997. The IPL gene on chromosome 11p15.5 is imprinted in humans and mice and is similar to TDAG51, implicated in Fas expression and apoptosis. *Hum. Mol.* *Genet.* **6**:2021–2029.

MacDonald, H. R. and Wevrick, R. 1997. The necdin gene is deleted in prader-willi syndrome and is imprinted in human and mouse. *Hum. Mol. Genet.* **6**:1873–1878.

Jay, P., Rougeulle, C., Massacrier, A., Moncla, A., Mattei, M. G., Malzac, P., Roeckel, N., Taviaux, S., Lefranc, J. L., Cau, P., Berta, P., Lalande, M., and Muscatelli, F. 1997. The human necdin gene, NDN, is maternally imprinted and located in the Prader-Willi syndrome chromosomal region. *Nat. Genet.* **17**:357–361

Driscoll, D. J., Waters, M. F., Williams, C. A., Zori, R. T., Glenn, C. C., Avidano, K. M., and Nicholls, R. D. 1992. A DNA methylation imprint, determined by the sex of the parent, distinguishes the Angelman and Prader-Willi syndromes. *Genomics* **13**:917–924

Glenn, C. C., Porter, K. A., Jong, M. T., Nicholls, R. D., and Driscoll, D. J. 1993. Functional imprinting and epigenetic modification of the human SNRPN gene. *Hum. Mol. Genet.* **2**:2001–2005.

Jong, M. T., Gray, T. A., Ji, Y., Glenn, C. C., Saitoh, S., Driscoll, D. J., and Nicholls, R. D. 1999. A novel imprinted gene, encoding a RING zinc-finger protein, and overlapping antisense transcript in the Prader-Willi syndrome critical region. *Hum. Mol. Genet.* **8**:783–793.

Boccaccio, I., Glatt-Deeley, H., Watrin, F., Roeckel, N., Lalande, M., and Muscatelli, F. 1999. The human MAGEL2 gene and its mouse homologue are paternally expressed and mapped to the Prader-Willi region. *Hum. Mol. Genet.* **8**:2497–2505.

Lee, S., Kozlov, S., Hernandez, L., Chamberlain, S. J., Brannan, C. I., Stewart, C. L., and Wevrick. 2000. Expression and imprinting of MAGEL2 suggest a role in Prader-willi syndrome and the homologous murine imprinting phenotype. *Hum. Mol. Genet.* **9**:1813–1819.

Rougeulle, C., Glatt, H., and Lalande, M. 1997. The Angelman syndrome candidate gene, UBE3A/E6-AP, is imprinted in brain. *Nat. Genet.* **17**:14–15.

Vu, T. H. and Hoffman, A. R. 1997. Imprinting of the Angelman syndrome gene, UBE3A, is restricted to brain. *Nat. Genet.* **17**:12–13.

Strichman-Almashanu, L. Z., Lee, R. S., Onyango, P. O., Perlman, E., Flam, F., Frieman, M. B., and Feinberg, A. P. 2001. A genome-wide screen for normally methylated human CpG islands that can identify novel imprinted genes. *Genome Res.* **12**:543–554

Evans, H. K., Wylie, A. A., Murphy, S. K., and Jirtle, R. L. 2001. The neuronatin gene resides in a “micro-imprinted domain” on human chromosome 20q11.2. *Genomics* **77**:99–104

Luedi, P.P., Dietrich, F.S., Weidman, J.R., Bosko, J.M., Jirtle R. L., Hartemink A. J. (2007) *Genome Res.;* **17**: 1723-1730

Mostoslavsky, R., Singh, N., Tenzen, T., Goldmit, M., Gabay, C., Elizur, S., Qi, P., Reubinoff, B. E., Chess, A., Cedar, H., and Bergman, Y. 2001. Asynchronous replication and allelic exclusion in the immune system. *Nature* **414**:221–225.

Kitsberg, D., Selig, S., Brandeis, M., Simon, I., Keshet, I., Driscoll, D. J., Nicholls, R. D., and Cedar, H. 1993. Allele-specific replication timing of imprinted gene regions. *Nature* **364**:459–463

Piras, G., El, K. h., Kozlov, S., Escalante-Alcalde, D., Hernandez, L., Copeland, N. G., Gilbert, D. J., Jenkins, N. A., and Stewart, C. L. 2000. Zac1 (Lot1), a potential tumor suppressor gene, and the gene for epsilon-sarcoglycan are maternally imprinted genes: identification by a subtractive screen of novel uniparental fibroblast lines. *Mol. Cell. Biol*. **20**:3308–3315.

Nabetani, A., Hatada, I., Morisaki, H., Oshimura, M., and Mukai, T. 1997. Mouse U2af1-rs1 is a neomorphic imprinted gene. *Mol. Cell Biol.* **17**:789–798.

Blin-Wakkach, C., Lezot, F., Ghoul-Mazgar, S., Hotton, D., Monteiro, S., Teillaud, C., Pibouin, L., Orestes-Cardoso, S., Papagerakis, P., Macdougall, M., Robert, B., and Berdal, A. 2001. Endogenous Msx1 antisense transcript: in vivo and in vitro evidences, structure, and potential involvement in skeleton development in mammals. *Proc. Natl. Acad. Sci*. **98**:7336–7341.

Bix, M. and Locksley, R. M. 1998. Independent and epigenetic regulation of the interleukin-4 alleles in CD4+ T cells. Science **281**:1352–1354

Buettner, V. L., Longmate, J. A., Barish, M. E., Mann, J. R., and Singer-Sam, J. 2004. Analysis of imprinting in mice with uniparental duplication of proximal chromosomes 7 and 15 by use of a custom oligonucleotide microarray. *Mamm. Genome* **15**:199–209

Kelly, B. and Locksley, R. 2000. Coordinate regulation of the IL-4, IL-13, and IL-5 cytokine cluster in Th2 clones revealed by allelic expression patterns*. J. Immunol*. **165**:2982–2986.

Takahashi, N. and Ko, M. 1993. The short 3’-end region of complementary DNAs as PCR-based polymorphic markers for an expression map of the mouse genome. *Genomics* **16**:161–168.

Kalscheuer, V. M., Mariman, E. C., Schepens, M. T., Rehder, H., and Ropers, H. H. 1993. The insulin-like growth factor type-2 receptor gene is imprinted in the mouse but not in humans. *Nat. Genet*. **5**:74–78.

Barlow, D. P., Stoger, R., Herrmann, B. G., Saito, K., Schweifer, N. B., Stoger, R., Herrmann, B. G., Saito, K., and Schweifer, N. 1991. The mouse insulin-like growth factor type-2 receptor is imprinted and closely linked to the Tme locus. *Nature* **349**:84–87

Schweifer, N., Valk, P. J., Delwel, R., Cox, R., Francis, F., Meier-Ewert, S., Lehrach, H., and Barlow, D. P. 1997. Characterization of the C3 YAC contig from proximal mouse chromosome 17 and analysis of allelic expression of genes flanking the imprinted Igf2r gene. *Genomics* **43**:285–297.

Wakeling, E. L., Abu-Amero, S. N., Stanier, P., Preece, M. A., and Moore, G. E. 1998. Human EGFR, a candidate gene for the Silver-Russell syndrome, is biallelically expressed in a wide range of fetal tissues. Eur*. J. Hum. Genet*. **6**:158–164.

Wakeling, E. L., Hitchins, M. P., Abu-Amero, S. N., Stanier, P., Moore, G. E., and Preece, M. A. 2000. Biallelic expression of IGFBP1 and IGFBP3, two candidate genes for the Silver-Russell syndrome. *J.* *Med. Genet.* **37**:65–67.

Hitchins, M. P., Bentley, L., Monk, D., Beechey, C., Peters, J., Kelsey, G., Ishino, F., Preece, M. A., Stanier, P., and Moore, G. E. 2002. DDC and COBL, flanking the imprinted GRB10 gene on 7p12, are biallelically expressed. Mamm. Genome **13**:686–691.

Eggermann, K., Wollmann, H. A., Binder, G., Kaiser, P., Ranke, M. B., and Eggermann, T. 1999. Biparental expression of IGFBP1 and IGFBP3 renders their involvement in the etiology of Silver-Russell syndrome unlikely. *Ann. Genet*. **42**:117–121.

Mizuno, Y., Sotomaru, Y., Katsuzawa, Y., Kono, T., Meguro, M., Oshimura, M., Kawai, J., Tomaru, Y., Kiyosawa, H., Nikaido, I., Amanuma, H., Hayashizaki, Y., and Okazaki, Y. 2002. Asb4, Ata3, and Dcn are novel imprinted genes identified by high-throughput screening using RIKEN cDNA microarray. *Biochem. Biophys. Res. Commun*. **290**:1499–1505.

Zhang, G., Taneja, K. L., Singer, R. H., and Green, M. R. 1994. Localization of pre-mRNA splicing in mammalian nuclei. *Nature* **372**:809–812.

Yamada, T., Mitsuya, K., Kayashima, T., Yamasaki, K., Ohta, T., Yoshiura, K., Matsumoto, N., Yamada, H., Minakami, H., Oshimura, M., Niikawa, N., and Kishino, T. 2003. Imprinting analysis of 10 genes and/or transcripts in a 1.5-mb mest-flanking region at human chromosome 7q32. *Genomics* **83**:402–412.

Bentley, L., Nakabayashi, K., Monk, D., Beechey, C., Peters, J., Birjandi, Z., Khayat, F. E., Patel, M., Preece, M. A., Stanier, P., Scherer, S.W., and Moore, G. E. 2003. The imprinted region on human chromosome 7q32 extends to the carboxypeptidase A gene cluster: an imprinted candidate for Silver-Russell syndrome*. J. Med. Genet*. **40**:249–256.

Walter, J. and Paulsen, M. 2003. The potential role of gene duplications in the evolution of imprinting mechanisms. *Hum. Mol. Genet*. **12**:215–220.

Singh, N., Ebrahimi, F. A., Gimelbrant, A. A., Ensminger, A. W., Tackett, M. R., Qi, P., Gribnau, J., and Chess, A. 2003. Coordination of the random asynchronous replication of autosomal loci. *Nat. Genet.* **33**:339–341.

Lerer, I., Sagi, M., Meiner, V., Cohen, T., Zlotogora, J., and Abeliovich, D. 2005. Deletion of the ANKRD15 gene at 9p24.3 causes parent-oforigin- dependent inheritance of familial cerebral palsy. *Hum. Mol. Genet*. **14**:3911–3920.

Lin, Z. and Floros, J. 2002. Heterogeneous allele expression of pulmonary SP-D gene in rat large intestine and other tissues. *Physiol. Genomics* **11**:235–243.

Paulsen, M., El-Maarri, O., Engemann, S., Strodicke, M., Franck, O., Davies, K., Reinhardt, R., Reik, W., and Walter, J. 2000. Sequence conservation and variability of imprinting in the Beckwith-Wiedemann syndrome gene cluster in human and mouse. *Hum. Mol. Genet*. **9**:1829–1841.

Maecker, H. T., Do, M. S., and Levy, S. 1998. CD81 on B cells promotes interleukin 4 secretion and antibody production during T helper type 2 immune responses. *Proc. Natl. Acad. Sci*. **95**:2458–2462.

Yuan, L., Qian, N., and Tycko, B. 1996. An extended region of biallelic gene expression and rodent-human synteny downstream of the imprinted H19 gene on chromosome 11p15.5. *Hum. Mol. Genet.* **5**:1931–1937.

Goldberg, M., Wei, M., Yuan, L., Murty, V., and Tycko, B. 2003. Biallelic expression of HRAS and MUCDHL in human and mouse. *Hum. Genet*. **112**:334–342.

Cost, G. J., Thompson, J. S., Reichard, B. A., Lee, J. Y., and Feinberg, A. P. 1997. Lack of imprinting of three human cyclin-dependent kinase inhibitor genes*. Cancer Res*. **57**:926–929.

Williamson, C. M., Dutton, E. R., Abbott, C. M., Beechey, C. V., Ball, S. T., and Peters, J. 1995. Thirteen genes (Cebpb, E2f1, Tcf4, Cyp24, Pck1, Acra4, Edn3, Kcnb1, Mc3r, Ntsr, Cd40, Plcg1 and Rcad) that probably lie in the distal imprinting region of mouse chromosome 2 are not monoallelically expressed. *Genet. Res*. **65**:83–93.

Yevtodiyenko, A., Carr, M. S., Patel, N., and Schmidt, J. V. 2002. Analysis of candidate imprinted genes linked to Dlk1-Gtl2 using a congenic mouse line. *Mamm. Genome* **13**:633–638

Chai, J. H., Locke, D. P., Greally, J. M., Knoll, J. H., Ohta, T., Dunai, J., Yavor, A., Eichler, E. E., and Nicholls, R. D. 2003. Identification of four highly conserved genes between breakpoint hotspots BP1 and BP2 of the Prader-Willi/Angelman syndromes deletion region that have undergone evolutionary transposition mediated by flanking duplicons*. Am. J. Hum. Genet*. **73**:898–925.

Farber, C., Gross, S., Neesen, J., Buiting, K., and Horsthemke, B. 2000. Identification of a testis-specific gene (C15orf2) in the Prader-Willi syndrome region on chromosome 15. *Genomics* **65**:174–183.

Chibuk, T. K., Bischof, J. M., and Wevrick, R. 2001. A necdin/MAGE-like gene in the chromosome 15 autism. *BMC Genet*. **2**.

Sandell, L. L., Guan, X. J., Ingram, R., and Tilghman, S. M. 2003. Gatm, a creatine synthesis enzyme, is imprinted in mouse placenta. *Proc. Natl. Acad. Sci*. **100**:4622–4627

Sano, Y., Shimada, T., Nakashima, H., Nicholson, R. H., Eliason, J. F., Kocarek, T. A., and Ko, M. S. 2001. Random monoallelic expression of three genes clustered within 60 kb of mouse t complex genomic DNA. *Genome Res*. **11**:1833–1841.

Amiel, A., Korenstein, A., Gaber, E., and Avivi, L. 1999. Asynchronous replication of alleles in genomes carrying an extra autosome. *Eur. J. Hum. Genet*. **7**:223–230.

Singh, N., Ebrahimi, F. A., Gimelbrant, A. A., Ensminger, A. W., Tackett, M. R., Qi, P., Gribnau, J., and Chess, A. 2003. Coordination of the random asynchronous replication of autosomal loci. *Nat. Genet.* **33**:339–341

Bonthron, D. T., Hayward, B. E., Moran, V., and Strain, L. 2000. Characterization of TH1 and CTSZ, two non-imprinted genes downstream of GNAS1
